# Microperimetry differences in macular sensitivity threshold between first and second tested eyes

**DOI:** 10.5935/0004-2749.20210034

**Published:** 2021

**Authors:** Natacha B. Junqueira, Luiz H. Lima, Rodrigo B. Ferreira, Denny Marcos Garcia, João M. Furtado, Rodrigo Jorge

**Affiliations:** 1 Department of Ophthalmology, Otorhinolaryngology and Head and Neck Surgery, Faculdade de Medicina de Ribeirão Preto, Universidade de São Paulo, Ribeirão Preto, SP, Brazil; 2 Department of Ophthalmology, Universidade Federal de São Paulo, São Paulo, SP, Brazil

**Keywords:** Macula lutea, Fixation, ocular, Bias, Visual field tests, Visual acuity, Macula lutea, Fixação ocular, Viés, Campos visuais, Acuidade visual

## Abstract

**Purpose:**

To comparatively assess the macular sensitivity threshold of microperimetry and the fixation stability between the first (right) and second (left) tested eye of normal participants.

**Methods:**

Thirty healthy patients were randomly assigned to two groups. The participants underwent microperimetry in the fast mode and expert mode in groups I and II, respectively. Each participant underwent a single test and the right eye was tested first.

**Results:**

The mean macular sensitivity threshold (± standard deviation [SD]) was 24.5 ± 2.3 dB and 25.7 ± 1.1 dB in the first (right) and second (left) eyes of group I, respectively (p=0.0415) and 26.7 ± 4.5 dB and 27.3 ± 4.0 dB in the first (right) and second (left) eyes of group II, respectively (p=0.58). There was no statistically significant difference between eyes in either group (p=0.1512). Regarding fixation stability (evaluated in the microperimetry expert mode group), the mean ± SD percentage of fixation points within the 1-degree central macula (P1) was 87.9 ± 11.5% in the right eye and 93.8 ± 6.6% in the left eye. The paired t-test did not show a statistically significant difference between eyes (p=0.140). Mean ± SD P2 value was 95.5 ± 4.9% in the right eye and 98.5 ± 2.1% in the left eye. The analysis demonstrated an increase in the percentage of fixation points in the second tested eye compared with the first one (paired t-test= 2.364; p=0.034). There was a negative correlation between the macular sensitivity threshold of the right eye and the duration of the examination for both groups (microperimetry expert mode: r=-0.717; p=0.0026; microperimetry in the fast mode: r=-0.843; p<0.0001).

**Conclusion:**

Mean macular sensitivity threshold was higher in the second tested eye in the microperimetry in the fast mode group and was similar in both eyes in the expert mode. Our data suggest that comprehension of the examination by the individual may impact the results of the microperimetry test.

## Abstract (pt)

**Objetivo:**

Avaliar comparativamente o limiar de sensibilidade macular da microperimetria e a estabilidade de fixação entre o primeiro (direito) e o segundo (esquerdo) olhos testados de indivíduos normais. **M**é**todos:** Trinta pacientes saudáveis foram divididos aleatoriamente em 2 grupos. Os participantes foram submetidos à microperimetria no “fast mode” e no “expert mode” no grupo I e II, respectivamente. Cada participante foi submetido a um único teste e o olho direito foi testado primeiro.

**Resultados:**

No grupo I, o limiar médio de sensibilidade macular (± DP) foi de 24,5 ± 2,3 dB e 25,7 ± 1,1 dB nos olhos direito e esquerdo, respectivamente (p=0,0415). No grupo II foi de 26,7 ± 4,5 dB e 27,3 ± 4,0 dB nos olhos direito e esquerdo, respectivamente (p=0,58). Não houve diferença estatisticamente significativa entre os olhos dos dois grupos (p=0,1512). Em relação à estabilidade de fixação (avaliada no grupo microperimetria no “expert mode”), a média das porcentagens dos pontos de fixação dentro do 1 grau central da mácula (P1) ± DP foi de 87,9 ± 11,5% no olho direito e de 93,8 ± 6,6% no olho esquerdo. O teste t pareado não mostrou diferença estatística entre os olhos (p=0,140). O valor médio de P2 ± DP foi de 95,5 ± 4,9% no olho direito e 98,5 ± 2,1% no olho esquerdo. Foi demonstrado um aumento na porcentagem de pontos de fixação no segundo olho testado quando comparado ao primeiro (teste t pareado= 2,364; p=0,034). Houve correlação negativa entre o limiar de sensibilidade macular do olho direito e a duração do exame nos dois grupos (microperimetria no “expert mode”: r=-0,717; p=0,0026; microperimetria no “fast mode”: r=-0,843; p<0,0001). **Conclus**ã**o:** O limiar médio de sensibilidade macular foi maior no segundo olho testado no grupo microperimetria no “fast mode” e foi semelhante nos dois olhos no “expert mode”. Nossos dados sugerem que a compreensão do exame pelo indivíduo pode impactar nos resultados da microperimetria.

## INTRODUCTION

Microperimetry, also termed fundus related perimetry, is a type of visual field test that creates a retinal sensitivity map of the amount of light perceived in specific parts of the retina^(1)^. Macular diseases typically result in deterioration of visual function that implies lower central macular sensitivity and fixation. In addition, visual acuity tests are unable to infer macular function because they do not permit the recognition of central or paracentral scotomas and the fixation changes that may strongly interfere with the patients’ quality of life^(2)^.

Microperimetry has been performed to determine the exact correlation between retinal diseases and functional defects, allowing the simultaneous observation of several visual field sites in the retinal fundus^(3)^. Luminous stimuli are presented to different areas of the visual field in order to be detected, and the patient must press a button when a luminous point appears. In this functional test, each site in the visual field has a sensitivity threshold defined as the weakest possible stimulus that can be observed at that site.

Heijl et al.^(4)^, demonstrated wide variation in the sequential visual field tests applied to individuals who were not familiar with the examination. This variability between eyes due to the learning curve may result in an erroneous interpretation that the second tested eye has a more preserved visual field than the first tested eye. Using microperimetry, Jones et al.^(5)^ observed variable sensitivity between examinations performed at different time points and recommended to discard the results of the first examination. However, in their report, it was not possible to evaluate the difference between eyes since only the dominant eye was tested. Furthermore, Barboni et al.^(6)^ tested both eyes of 12 healthy volunteers thrice and reported absence of significant variation in mean macular sensitivity. The purpose of the present study was to perform and comparatively assess the central macular sensitivity in the fast (ACF) and expert (ACE) modes and the fixation stability in the ACE in the first (right) and second (left) tested eyes of the same patient using microperimetry.

## METHODS

### Patients

This was a cross-sectional study in which the ACF and ACE modes of the Macular Analyzer Integrity Assessment (MAIA) microperimeter (CentreVue, Padova, Italy)^(7)^ were used for the analysis. The microperimetry examination was performed by one of the authors (RBF) of this study from the Department of Ophthalmology, Ribeirão Preto Medical School, University of São Paulo (Ribeirão Preto, Brazil) between February and April 2018. The study was approved by the Institutional Review Board of the University of São Paulo, Ribeirão Preto Medical School (reference number: 80069717.2.0000.5440) and written informed consent was obtained from all participants.

Individuals accompanying patients at the ophthalmology outpatient clinic were invited to participate in the study. Thirty individuals were randomly selected and subjected to microperimetry: 15 underwent examination using the ACE mode (ACE group) and the remaining 15 were analyzed using the ACF mode (ACF group). The subjects were tested once, and the examination was performed first in the right eye followed by the left eye. The inclusion criteria were: patients aged ≥18 years; best-corrected visual acuity of 20/20 or better; highest refractive error of ± 5.00 spheric and/or -2.00 cylinder; undilated pupils with a diameter ≥4 mm; and patient consent to perform the examination. The exclusion criteria were: ocular disease that may interfere with macular sensitivity; presence of myosis; and inability to understand the microperimetry examination.

### Examination

Microperimetry, such as standard automated perimetry, measures retinal sensitivity as the minimum light intensity that patients can perceive when spots of light stimulate specific areas of the retina. The standard MAIA examination covers a 10° diameter area with 37 measurement points using values of 27, 25, and 16 decibel (dB) for the green, yellow, and red colors, respectively. The stimulus size is Goldmann III, the background luminance is 4 asb and the maximum luminance is 1,000 asb with a 36 dB dynamic range.

The MAIA microperimeter permits different types of exa mination: 1) ACF mode (2-3 min per eye): suprathreshold examination with 37 stimuli for a rapid assessment of macular sensitivity measuring two levels of sensitivity (27 and 25 dB); and 2) ACE mode (4-7 min per eye): a full threshold examination with 37 stimuli used to examine retinal sensitivity in detail. This mode performs a complete assessment, determining macular threshold sensitivity and fixation stability, and measuring fixation stability by the percentage of fixation points located within a distance of 1° and 2° from the center of the fovea, respectively (P1 and P2).

The study data were compared between eyes according to the mean macular sensitivity. In addition, fixation stability was assessed by calculating the mean P1 and P2 values (ACE mode). Best-corrected visual acuity was measured using the Snellen chart placed at a distance of 4 m from the participant.

### Statistical analysis

Statistical analysis was performed using the JMP SAS 10.0 software (SAS Institute, Cary, NC, USA). The demographic characteristics of the participants were assessed by the Student’s t-test and chi-squared test. Two-factor mixed-design analysis of variance (ANOVA) was used for comparison between groups and between eyes for macular sensitivity threshold and duration. The Student’s t-test for paired samples was employed to test the fixation stability in 1- and 2-degree areas between eyes in the ACE group. The relationship between the macular sensitivity threshold and duration was analyzed using Pearson’s correlation coefficient. A p-value of 0.005 denoted a statistically significant difference.

## RESULTS

The ACE group consisted of nine women (60%) and six men (40%) aged 39.5 ± 11.7 (mean ± standard deviation [SD]) years (range: 19-55 years). The ACF group consisted of 12 women (80%) and three men (20%) aged 39.2 ± 12.5 years (range: 21-70 years). Data analysis showed that there was no significant difference in age (p=0.95) or sex (p=0.23) between the study groups.

In the ACF group, the mean macular sensitivity threshold (± SD) was 24.5 ± 2.3 dB and 25.7 ± 1.1 dB in the first (right) eye and second (left) eye of participants, respectively. In the ACE group, the mean macular sensitivity threshold (± SD) was 26.7 ± 4.5 dB and 27.3 ± 4.0 dB, respectively (Table 1).

**Table 1:** Mean macular sensitivity threshold (dB) in the right and left eyes from the ACE and ACF groups

Group	Right eye				Left eye			
	Mean ± SD	Min	Med	Max	Mean ± SD	Min	Med	Max
ACE	26.7 ± 4.5	15.3	28.2	31.7	27.3 ± 4.0	17.6	28.3	32.1
ACF	24.5 ± 2.3	19.9	25.0	26.8	25.7 ± 1.1	23.6	26.0	26.8

ACE= subjects who underwent microperimetry in the expert mode; ACF= subjects who underwent microperimetry in the fast mode; SD= standard deviation; Min= minimum; Med=median; Max= maximum.

**Figure 1:**
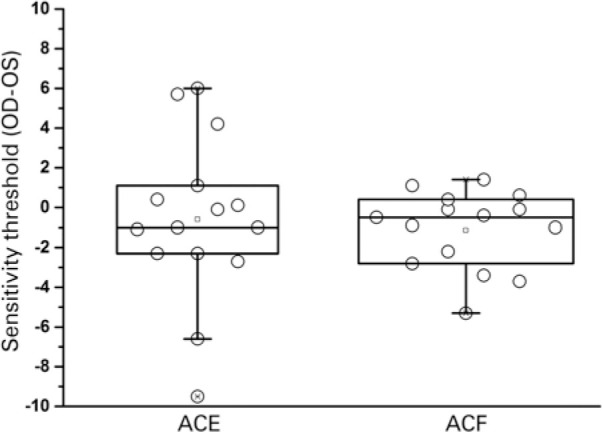
Box plots showing the results for the difference in mean macular sensitivity threshold between the first (right: OD) and second (left: OS) eyes in each group for the fast mode (ACF) and expert mode (ACE) microperimetry evaluation modalities. The middle line represents the median, the square represents the mean, 25^th^, and 75^th^ percentiles determine the box, and the 5th and 95th percentiles determine the whiskers.

Two-factor mixed-design ANOVA did not reveal a significant difference in the macular sensitivity threshold between eyes (within-subject factor, p=0.1512), study groups (between-subjects, p=0.0684) or eye-group interaction (p=0.6614) (Figure 1). When the groups were analyzed separately, the ACF group showed a significant difference in the macular sensitivity threshold between eyes (paired t-test: t=2.24; p=0.0415). There was no statistical difference observed in the ACE group (paired t-test: t=0.57; p=0.58).

ACE= subjects who underwent microperimetry in the expert mode; ACF= subjects who underwent microperimetry in the fast mode; OD= oculus dexter; OS= oculus sinister.

Fixation stability was analyzed only in the ACE mode. The mean percentage of fixation points in the 1-degree central macula (± SD) was 87.9 ± 11.5% in the right eye and 93.8 ± 6.6% in the left eye, without difference between eyes (paired t-test= 1.570; p=0.140). The mean percentage of fixation points in the 2-degree central macula (± SD) was 95.5 ± 4.9% in the right eye and 98.5 ± 2.1% in the left eye, showing an improvement in the second tested eye compared with the first tested one (paired t-test= 2.364; p=0.034) (Tables 2 and 3).

**Table 2:** P1: percentage of fixation points located within a distance of 1°

Group	Right eye				Left eye			
	Mean ± SD	Min	Med	Max	Mean ± SD	Min	Med	Max
ACE	87.9 ± 11.5	59.0	92.0	100.0	93.8 ± 6.6	77.0	94.5	100.0
ACF	-	-	-	-	-	-	-	-

ACE= subjects who underwent microperimetry in the expert mode; ACF= subjects who underwent microperimetry in the fast mode; SD= standard deviation; Min Med, median; Max= maximum.

**Table 3:** P2: percentage of fixation points located within a distance of 2°

Group	Right eye				Left eye			
	Mean ± SD	Min	Med	Max	Mean ± SD	Min	Med	Max
ACE	95.5 ± 4.9	86.0	97.0	100.0	98.5 ± 2.1	93.0	99.5	100.0
ACF	-	-	-	-	-	-	-	-

ACE= subjects who underwent microperimetry in the expert mode; ACF= subjects w Med= median; Max= maximum.

**Figure 2:**
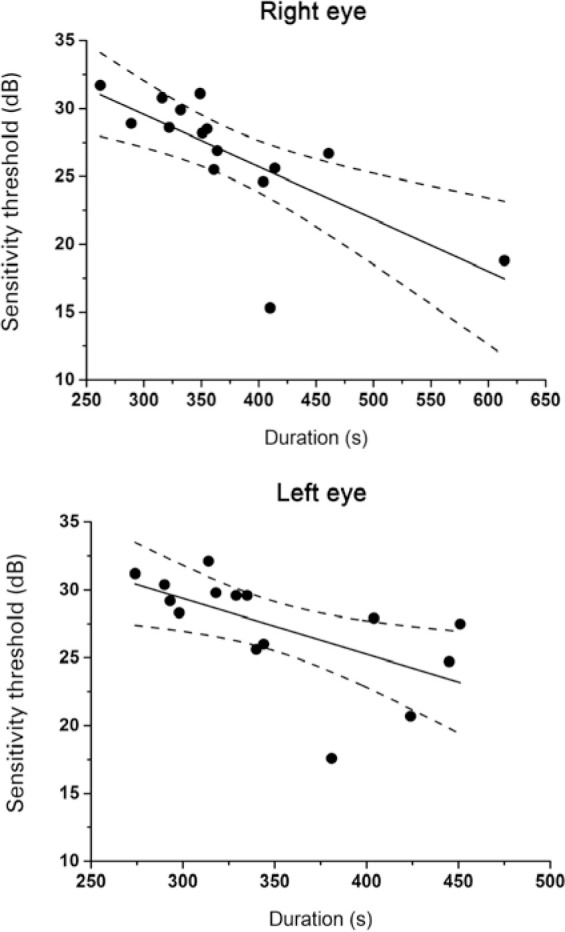
Macular sensitivity threshold as a function of examination duration for the first (right) and second (left) eye, respectively - ACE group.

The two-factor mixed-design ANOVA revealed a significant difference in duration between the groups (between-subjects, p<0.001), but not between eyes (within-subject factor, p=0.4520) or interaction (EYE*GROUP, p=0.5186) (Table 4). There was a negative correlation between the macular sensitivity threshold of the right eye and the duration of the examination for both groups (ACE: r=-0.717; p=0.0026; ACF: r=-0.843; p<0.0001), indicating that a longer duration led to lower thresholds. Regarding the left eye, there was a significant correlation noted only for the ACE group (ACE: r=-0.604; p=0.0171; ACF: r=-0.3499; p=0.201) (Figure 2).

**Table 4:** Duration of the examinations (s)

Group	Right eye			Left eye			
	Mean ± SD	Min Med	Max	Mean + SD	Min	Med	Max
ACE	373.6 ± 83.8	262.0 355.0	614.0	349.3 ± 58.0	274.0	335.0	451.0
ACF	146.7 ± 39.2	110.0 139.0	232.0	144.8 ± 70.5	93.0	134.0	392.0

ACE= subjects who underwent microperimetry in the expert mode; ACF= subjects who underwent microperimetry in the fast mode; SD= standard deviation; Min= minimum; Med= median; Max= maximum.

ACE= subjects who underwent microperimetry in the expert mode.

## DISCUSSION

Fundus view in real time during microperimetry allows the positioning of stimuli at any point of the central retina area. By using this technology, structure-function associations can be determined, as well as direct correlations with other retinal examination modalities, such as fundus autofluorescence and optical coherence tomography^(8)^. Furthermore, a more rapid acquisition, in addition to these benefits, is an advantage of microperimetry over conventional perimetry^(9)^.

Previous studies have observed increased sensitivity between consecutive examinations due to the learning effect. Wu et al.^(10)^ demonstrated significant evidence of learning between the first and second examinations. To avoid biases and increase accuracy, the present study comparatively assessed the results for both eyes of the same patient in a single examination. In accordance with other studies that evaluated the improvement in serial microperimetry evaluations, we observed that the patients showed better results from one eye to the other already during the first microperimetry evaluation. This feature was highlighted in the ACF group that showed difference between eyes in the macular sensitivity threshold, demonstrating that the learning curve may occur during the execution of the examination. These data suggest that the learning factor interferes with the results of the examination, a fact that should be considered in data analysis.

The absence of a statistically significant difference in the macular sensitivity threshold between eyes in the ACE group may be explained by the longer duration of the examination, allowing improvement of the score during the first eye test. In other words, faster tests lower the patient’s ability to learn the examination in the first eye test. For this reason, the examination results of the first tested eye should be analyzed with caution.

The values of the mean macular sensitivity threshold found in this study (ACF: 25.1dB; ACE: 27 dB) were lower than the normative value of 29.8 dB^5^ and those reported in other studies: 33 dB for patients aged 21-50 years^(11)^, 28.52 dB^(12)^, >28 dB^(6)^, and 30.68 dB^(8)^.

Furthermore, in the ACE group, examinations with shorter duration were associated with higher mean sensitivity thresholds. This association was not observed in the ACF group due to the short duration of this examination modality. Our experience with microperimetry revealed that this difference is due to reduced patient concentration throughout the examination. Hudson et al.^(13)^ and Balasubramanian et al.^(8)^ observed that perimetric examination and microperimetry can be tiring for the patient. Moreover, it is possible that, with a longer test, patients may have experienced more fatigue, negatively affecting their performance.

Regarding fixation stability, Morales et al.^(9)^ demonstrated higher P1 values than those found in our study (95% vs. 90.85%, respectively), while the P2 index showed a smaller difference (99% vs. 97%, respectively). The median P1 and P2 values reported by Molina-Martín et al.^(11)^ were 98.00% and 100.00% (1.00), respectively. However, the present study showed an improvement in the percentage of fixation points in the 2-degree central macula in the second tested eye compared with the first tested one. This evidence was not reported in other studies.

On the basis of the present data, we conclude that the microperimetry examination showed biases, such as patient concentration during the examination and patient learning, as determined by the analysis of the correlation between the duration of the examination and macular sensitivity and by the comparison of the sensitivity between eyes. These conditions should be considered in the interpretation of microperimetry results.
